# 10-kHz High-Frequency SCS Therapy: A Clinical Summary

**DOI:** 10.1111/pme.12617

**Published:** 2014-11-07

**Authors:** Marc Russo, Jean-Pierre Van Buyten

**Affiliations:** * Hunter Pain ClinicBroadmeadow, New South Wales, Australia; † Multidisciplinary Pain Center, AZ NikolaasSint-Niklaas, Belgium

**Keywords:** Axial Back Pain, Low Back Pain, Chronic Pain, Failed Back Surgery Syndrome, High-Frequency Stimulation, Spinal Cord Stimulation, HF10

## Abstract

**Objective:**

Chronic pain remains a serious public health problem worldwide. A spinal cord stimulation (SCS) therapy called HF10 SCS uses 10-kHz high-frequency stimulation to provide pain relief without paresthesia. In this article, we describe the therapy, device, and the methods of implant and then review the safety and effectiveness data for this therapy.

**Results:**

HF10 SCS uses a charge-balanced stimulation waveform that has been shown to be safe in both animal and human studies. Data from a multicenter, prospective clinical trial shows that the therapy provides substantial back and leg pain relief. Numerous additional reports suggest improved pain relief in other body areas and for complex pain patterns, even for patients who have previously failed other neuromodulation therapies.

**Conclusions:**

The clinical experience reported in this article supports the efficacy and pain relief provided by HF10 SCS therapy. Clinical studies have also concluded that HF10 SCS does not generate paresthesia nor was it necessary to provide adequate coverage for pain relief. As clinical evidence accumulates and technological innovation improves patient outcomes, neuromodulatory techniques will be sought earlier in the treatment continuum to reduce the suffering for the many with otherwise intractable chronic pain.

## Background

Chronic pain remains a serious public health problem. A recent survey estimates that almost one in five (19%) adults in Europe suffer from chronic pain with almost two-thirds of chronic pain patients reporting inadequate pain control at times [1]. The economic burden associated with chronic pain is substantial. For example, an analysis by Maniadakis and Gray [2] showed that in the UK, back pain, with an annual indirect cost exceeding £10 billion, imposed a greater economic burden than any other disease for which an economic analysis has been calculated. A recent report by the Institute of Medicine estimates that chronic pain affects approximately 100 million adults in the United States with a cost of $560–635 billion annually. Other countries show similar high numbers of prevalence and cost burdens [3].

Spinal cord stimulation (SCS) is an accepted cost-effective treatment option for chronic pain 4–7. SCS is typically recommended after conventional medical management and/or back surgery has failed. SCS has the advantages of being reversible and less invasive than surgeries and may cause fewer issues over time than long-term pharmacological treatments, therefore causing some to call for its use earlier in the treatment continuum [8].

Nevertheless, traditional SCS therapy still has limitations. The objective of traditional SCS is to induce comfortable paresthesia, tingling sensations, that overlap the existing distribution of pain by modulating neuronal activity [9]. The success of traditional SCS in relieving pain has been correlated to the coverage of paresthesia over the painful body area(s), as well as patient acceptance of the induced sensations [10]. Creating the desired paresthesia coverage can be difficult, particularly for back pain patients [11].

Some patients find SCS-induced paresthesia to be uncomfortable. Furthermore, paresthesia variability can also increase with postural changes and body movement to the point of discomfort or in other cases diminishing to subtherapeutic levels [12]. Obtaining and maintaining paresthesia over certain areas of pain and/or treating complex pain can be difficult [13]. Many technological advances have been introduced to address these shortcomings of SCS therapy, primarily seeking to provide better paresthesia overlap of difficult body targets, such as the low back 10,14–19, but with limited success 11,20–23. Providing pain relief for these hard-to-treat areas remains a challenge.

An SCS system capable of delivering stimulation frequencies up to 10 kHz has been developed (Senza® SCS system, Nevro Corp, Menlo Park, CA, USA). It delivers HF10™ SCS therapy, a therapy that uses proprietary waveform with stimulation frequencies up to 10 kHz. In contrast to other currently available systems that use frequencies in the range of 50 Hz, this innovation does not require or produce paresthesia to achieve clinical efficacy [24]. Multicenter, prospective, open-label studies in patients in the United States [24] and the EU [25] have been conducted, in which evidence has been generated suggesting safety and long-lasting pain relief of HF10 SCS therapy.

Since its European regulatory approval in 2010, the Senza system has been implanted in over 2,000 patients. The device is approved in Europe and Australia for the management of chronic intractable pain of the trunk and/or limbs and is currently in preapproval clinical trials in the United States. In this report, we summarize the device, the implanting and programming procedures, as well as review the available clinical safety and effectiveness data. Lastly, implications on future pain management practice are discussed. The focus of this report is to provide a descriptive summary of an approved device and an up-to-date review of the current published clinical experience, rather than to address mechanisms of action, or comparative efficacy with other treatment modalities.

## Overview of HF10 SCS Therapy

HF10 SCS is SCS provided at a much higher frequency than traditional SCS systems. The HF10 SCS waveform consists of a biphasic charge-balanced pulse train with pulse widths usually set to 30 μsec and a pulse rate of 10 kHz. Table 1 shows a comparison of HF10 SCS with traditional SCS.

**Table 1:** Comparison of HF10 SCS with traditional SCS

System	HF10 SCS	Traditional SCS
Typical pulse width (μsec)	30	400
Typical stimulation rate (Hz)	10,000	40
Typical stimulation location for back pain	T9-T10	T8
Typical stimulation location for neck and arm pain	C2-C4	C2-C7
Typical amplitude for back pain (mA)	1–5	4–6
Implant procedure	Leads placed by anatomical landmarks Patient under continual sedation	Leads placed based on verbal patient feedback Patient provides feedback on paresthesia coverage Intraoperative programming and lead repositioning often required
Stimulation trial	Clinical goal is to reduce pain	Clinical goal is to reduce pain by achieving technical goal (cover pain with paresthesia)

SCS = spinal cord stimulation.

### Implant Procedure

Under fluoroscopic guidance, stimulation leads are placed according to anatomical landmarks. Leads are placed in the midline epidural space with a staggered offset to cover the target areas in a contiguous fashion. For back and leg pain, the first electrode of one lead is usually placed at the top of the T8 vertebra and the last electrode of the second lead placed at the bottom of the T11 vertebra with some overlap of the leads at the T9/T10 disc. For neck and arm pain, the electrodes are placed along the C2–C7 vertebrae. These positions have been determined by empirical observations to be effective. The overlap provides some protection against lead migration, which can be especially common during the trial phase. This large span is provided by SCS octopolar leads that have 5-mm spacing between the contact edges. The anatomy-based surgical placement simplifies the implant procedure compared with traditional SCS, where the physician must take time to position the leads to assure that stimulation from the electrodes covers the patient's painful areas with paresthesia. This process often referred to as “paresthesia mapping” involves changing sedation and verbally interacting with the patient to discuss perceived sensations. The physician often must physically move the leads and perform intraoperative programming with the aid of a technician to optimize paresthesia distribution and comfort. With HF10 SCS, because the physician does not need to check for paresthesia coverage, the patient is allowed to remain under sedation continuously, improving surgical workflow and increasing patient comfort. After the leads are placed, the impedances are checked to ensure the integrity of the stimulation system, and the leads are verified to be in the epidural space before completing the procedure.

### Trial Phase and Programming

With HF10 SCS therapy, the patient undergoes a stimulation trial similar to traditional SCS. The leads are externalized and connected to an external trial stimulator for a period ranging from a few days to a few weeks depending on the local standard of care. Patients are provided three programs initially to evaluate which program provides the best pain relief. These programs involve multiple contacts and typically utilize amplitude between 1 and 5 mA, with amplitude gradually increased if no pain relief is experienced. A program is generally tried for a period of 24 hours and continued if successful analgesia is obtained or changed to a different program if insufficient analgesia is obtained. If none of the three programs is found to provide relief, the patient will return to the clinic for another set of programs. A permanent implantable pulse generator (IPG) is typically implanted if the trial of HF10 provided greater than 50% pain relief.

### Patient Follow-Up

Patient follow-up is similar to traditional SCS, with patients coming in every 3 months to 1 year depending on the physician's usual practice. As in traditional SCS, patients are given instructions that if their pain relief changes or additional issues arise, they should return to the clinic for evaluation and potential reprogramming or lead revision. Clinical evaluation may include assessment of new pain, and a technical assessment may include impedance testing and x-rays to determine if the leads have migrated or if some of the contacts are not functioning, similar to traditional SCS.

Overall, as summarized in Table 1, the Senza system with HF10 SCS may provide substantial benefits over traditional SCS systems.

## Clinical Data Summary

The following sections review the clinical evidence collected to date on the Senza system and HF10 SCS therapy.

### Safety Data

The Senza device has been extensively reviewed and deemed safe by regulatory agencies and has since been approved for use in Europe and Australia. This section will summarize the system's design as it relates to safety and clinical safety results.

The Senza system has been designed to deliver a safe waveform—a current-controlled pulse delivered at 10,000 Hz. The waveform maintains charge balance, an important aspect of neurostimulation safety, through the use of “active charge recovery.” With the Senza system, the current is “pushed out” then “pulled in” by the electronic circuits, where both phases are actively controlled in a precise manner. Active charge recovery can allow the charge balance to happen much more quickly and controllably than “passive recharge” where the current is pushed out and is then passively recovered, as is the case with traditional low-frequency SCS therapy. Active recharge allows the stimulation frequency to reach the high rates used in HF10 SCS while still maintaining charge balance, important for safely delivering electrical pulses to the tissues.

To test for safety, Butt et al. [26] used HF10 SCS stimulation in a caprine model. Twelve goats were implanted; half received HF10 SCS, while the others received no stimulation. The animals were stimulated continuously for 10 days. The duration was selected because stimulation-induced tissue damage is preceded by detectable neurohistologic changes that happens well within 240 hours [27]. After this time, no histological differences between the two groups could be discerned by a blinded veterinary pathologist, indicating that the stimulation was well tolerated.

More recent safety data comes from a prospective, multicenter, open-label clinical trial at two European centers: Department of Anesthesia and Pain Management, AZ Nikolaas, St Niklaas, Belgium, and The Pain Management and Neuromodulation Centre, Guy's and St. Thomas’ Hospital, London, UK [25],[28]. In this study, 83 subjects with significant back pain were recruited, and 72 received a permanent implant. Most subjects (N = 65) were then followed for 24 months. The serious adverse device-related events (Table 2) were consistent with those noted for traditional SCS by Bendersky and Yampolsky [29], with the most common being pain at the IPG pocket site, wound infection, and lead migration.

**Table 2:** Serious adverse device events at 2-year follow-up

SADEs	Number of Events	% of Patients
Pocket pain	7	8.4
Wound infection	5	6.0
Lead migration	4	4.8
Loss of therapy effect	2	2.4
Suboptimal lead placement	1	1.2
Skin erosion	1	1.2

SADE = serious adverse device-related event.

In addition to collection of adverse events, the centers performed neurologic exams at baseline and at study follow-ups. This data were reviewed by an independent panel of neurologists. No evidence of neurologic deficit or dysfunction related to the stimulation was observed.

Overall, this long-term data demonstrates that the Senza system's safety profile is similar to that of traditional SCS and that when adverse events do occur, they are generally mild and reversible.

### Effectiveness Data

The effectiveness of the Senza system can be evaluated by assessing the outcomes from two prospective multicenter studies, as well as a number of smaller patient cohorts. The most compelling results originate from patients with back and leg pain, but there are also limited data on patients with other conditions, including complex regional pain syndrome (CRPS).

The first clinical study of HF10 SCS was a feasibility study in 24 patients at five U.S. centers with chronic predominant back pain who were candidates for traditional SCS [24]. Patients received a 4-day trial of HF10 SCS after receiving 4 days of a trial with traditional SCS. Compared with baseline, patients receiving HF10 SCS in the study showed significant improvement in overall visual analog scale (VAS) pain scores (8.68–2.03 [*P* < 0.001]) and back pain scores (8.12–1.88 [*P* < 0.001]) with HF10 SCS. The vast majority (21 out of 24, 87.5%) of patients preferred HF10 SCS therapy over conventional therapy.

**Figure 1:**
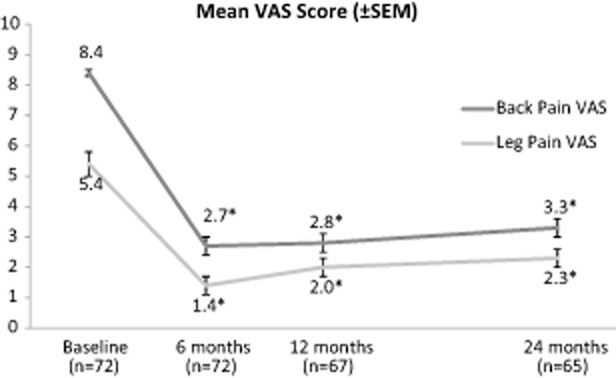
Back and leg visual analog scale (VAS) scores, change from baseline by visit with ± standard error of the mean from the European study. * *P* value <0.001 compared with baseline.

As just described, efficacy results are also available from a prospective, multicenter, open-label clinical trial for patients with chronic intractable back pain with 2-year follow-up conducted in Europe [25],[28]. In this study, the patient's back and leg pain VAS, Oswestry Disability Index (ODI), and opioid intake were recorded. The VAS scores are shown in Figure 1. Shown alongside the outcomes from the two randomized controlled SCS trials [30–32] are the main results of the study summarized in Table 3. For the outcome measures studied, patients with HF10 SCS demonstrated clinically and statistically meaningful improvement. Additionally, the improvement was larger than the results reported in the other SCS studies, indicating that HF10 SCS may provide pain relief beyond what is usually seen in traditional SCS.

**Table 3:** Results from large FBSS SCS studies with long-term follow-up

				Leg Pain (VAS Score, Responders)		Back Pain (VAS Score, Responders)		Function (ODI Score)		Opioids (Points on Opioids, mg/day)	
Study	Main Pain Area	Trial Success, # of Points (%)	# of Points at 24 Months	Baseline	24 Months	Baseline	24 Months	Baseline	24 Months	Baseline	24 Months
EU study	Back	72/82 (88)	65	5.4	2.3*	8.4	3.3*	55	40*	86%	57%*
					71%		60%			84	27*
North et al.	Leg	17/24 (71)	19	NR	NR	3.3	NR	NR	NR	NR	NR
					47%‡		NR	NR	NR	NR	NR
Kumar et al.	Leg	43/52 (83)	42	7.6	4.4*	5.5	4.8†	55	46*	71%	62%†
					40%		NR			81	83†

PROCESS study's 2-year VAS and ODI scores are estimated from charts [31].

* Statistically significant compared with baseline.

† Not statistically significant compared with baseline.

‡ At follow-up of 2.9 ± 1.1 years.

FBSS = failed back surgery syndrome; NR = not reported; ODI = Oswestry Disability Index; SCS = spinal cord stimulation; VAS = visual analog scale.

**Figure 2:**
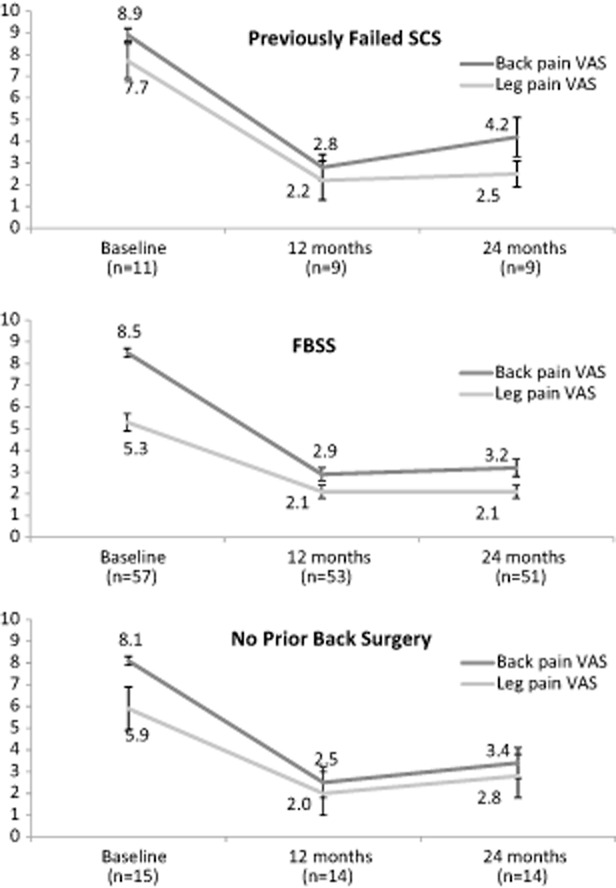
Back and leg visual analog scale (VAS) scores, change from baseline by visit for previously failed spinal cord stimulation (SCS) and failed back surgery syndrome (FBSS), and no prior back surgery patient subsets from the European study.

In this study, patients were also evaluated in subpopulations based on their clinical history [25],[28]. Results were analyzed for patients with failed back surgery syndrome, patients with no prior back surgery, and patients who had previously failed SCS therapy. All three subgroups showed results similar to the overall cohort, indicating that this therapy tends to provide relief for most types of chronic back and/or leg pain regardless of medical history. The VAS pain reduction scores for these patients can be seen in Figure 2 [33–35].

Another post hoc subgroup analysis presented by Smet and colleagues [36] showed that leg pain was substantially reduced (similar to back pain reduction) in patients in the European study. Most of the patients (49 out of 83) in the EU study had leg pain scores as well as back pain scores that were greater than 5 out of 10 at baseline. The vast majority of these (43 out of 49) had successful pain reductions during their trial (greater than 50%) and received a permanent HF10 SCS system. These patients showed a 74% reduction in mean leg VAS at 6 months compared with baseline (*P* < 0.001) and a 62% reduction at 12 months (*P* < 0.001). Most patients (65%) had at least 50% leg pain relief at 12 months compared with baseline. Back pain was also reduced, with a 62% reduction at 12 months compared with baseline, indicating that HF10 SCS can simultaneously improve leg pain and back pain.

In addition to the formal clinical studies, data from four [4] Australian centers involving 297 HF10 SCS trial procedures were reviewed [37]. Data included patients with pain in the following areas: back and leg (N = 136), back only (N = 50), head with or without neck (N = 21), neck with or without arm/shoulder (N = 15), as well as other complex pain patterns. Of these patients, 25% had failed conventional SCS or peripheral nerve field stimulation (PNFS). In general, data were collected by physicians at baseline, after the trial, and post-implant as per each site's practice. Of the 297 patients trialed, 220 (74%) had a positive trial and proceeded to implant. Patients with predominant back pain and concomitant leg pain demonstrated the highest trial success rate (82%). Of the patients that had previously failed conventional SCS and/or PNFS, the vast majority (69%) had a positive HF10 SCS trial. Patients showed a 4.8 ± 1.9 overall pain reduction measured using the numerical rating scale (NRS; 0–10) (7.4 ± 1.6 vs 2.6 ± 1.7 *P* ≤ 0.001) from baseline to post-trial (N = 172) as well as 3.6 overall NRS reduction (7.4 ± 1.6 vs 3.7 ± 2.1, *P* ≤ 0.001) from baseline to 12 months post-implant (N = 58). The NRS for back pain was collected in a subset of patients with back pain only (N = 30) and showed a reduction from 7.1 ± 1.7 at baseline to 3.3 ± 1.8 at 3 months and 3.2 ± 2.2 at 6 months (*P* < 0.001), with the results being similar to those published for the European study [25].

Combined, these results provide evidence that HF10 SCS therapy is able to provide effective relief for many patients with chronic back, leg, and upper body pain due to various underlying diagnoses.

### Additional Studies

In addition to the larger studies, a number of single centers have confirmed that HF10 provides relief of chronic pain under real-world conditions and in a wider variety of pain conditions through retrospective data analyses.

Al-Kaisy and colleagues [38] recently presented results on HF10 SCS for the treatment of patients with chronic neuropathic limb pain, including patients with CRPS of the hand or foot and postsurgical knee pain. Leads were placed at the level of the cervical or thoracic spine, depending upon the painful body region. Fifteen patients were trialed, and 11 proceeded to permanent implant. Seventy-three percent of patients (8/11) had greater than 50% reduction in pain relief at 6 months, and all but one patient had good or excellent satisfaction with their treatment. Similarly, Wohak [39] presented results from three CRPS patients with pain in the upper extremities. These patients also had leads placed at the level of the cervical spine. All three patients had significant reductions in their pain scores and recovery of function of their upper limbs. These results suggest that HF10 SCS may provide relief of other forms of chronic neuropathic pain beyond that of the back and legs.

Verrills et al. report on a cohort of chronic back pain patients who failed traditional SCS [40] and those who failed PNFS [41]. In both cases, the improvement in pain relief was substantial, with pain NRS reduced from a mean of 7.1 ± 1.7 prior to the HF10 trial to 3.1 ± 1.8 after the HF10 trial for failed SCS patients, and from a mean of 6.5 ± 1.8 prior to the HF10 trial to 2.4 ± 1.6 after the HF10 for the failed PNFS patients. There were trends toward improvement in patient medication use, ODI, ability to function, and psychological state for both patient groups. This result confirms the European study subgroup analyses that patients who failed traditional neurostimulation therapies may still succeed with HF10 SCS.

Finally, Salmon [42] showed outcomes for 79 consecutive patients with a number of conditions, including back, leg, and/or neck pain following surgery, chronic migraine, and whole-body neuropathic pain syndromes. Many of the patients had previously failed traditional SCS and/or PNFS (55%). Of the 79 patients, 60 (75%) had a successful trial. Of those, 54 were followed for an average of 9 months (range 4–19 months); two patients died due to reasons unrelated to the therapy or procedure, and another two were explanted due to infection. The NRS pain scores decreased from 7.8 ± 1.3 at baseline to 4.0 ± 2.1 at the time of the last follow-up visit. All of the different pain etiology groups showed improvement. Of the 54 patients, 34 patients had pretreatment questionnaires that were suitable for postimplant comparisons. These patients were mailed follow-up questionnaires, and 29 (79%) were returned. Of these, 58% of the 12 patients taking strong opiates reduced their dosage by an average of 44%, and of seven patients with intrathecal pumps, 57% ceased use or reduced dosage by more than 50%. This real-world, consecutive cohort of patients with a variety of pain conditions, in conjunction with the other smaller studies summarized here, provides further evidence that HF10 SCS is a useful therapeutic option for a wide range of chronic pain patients.

## Discussion

This report describes recent experience with the HF10 SCS to treat chronic pain. HF10 SCS has been shown to address pain areas that traditional SCS therapy is known to be less effective in treating, including the low back [13]. This therapy also shows promise for patients with CRPS and patients with intractable back pain who have not undergone back surgery.

This therapy overcomes many of the known limitations of traditional SCS therapy. In particular, HF10 SCS does not require the patient to feel paresthesia, which some patients may find uncomfortable [12]. Kuchemann reported that 71% of patients with traditional SCS experience uncomfortable paresthesia when changing position. A recent study [19] reported that an accelerometer-based solution to improve these variations with position (RestoreSensor®, Medtronic, USA) has reduced this problem, but 19.7% still felt “no comfort during position changes,” and 52.1% had no improvements in sleep. HF10 SCS's lack of paresthesia means no painful stimulation during positional changes so that patients need not interrupt therapy during sleep and while driving, perhaps improving the patient's quality of life and engagement of normal activities.

One of the most compelling aspects of this therapy is that the pain relief results obtained are at least as favorable as traditional SCS therapy (see Table 3) and are sustained over time. Although contribution of a placebo effect is possible in any intervention, the fact that substantial and significant pain relief was maintained for 24 months contributes to the evidence of a true therapeutic effect. Moreover, although the clinical evidence obtained to date has been from longitudinal interventional studies without concurrent controls, the magnitude and durability of pain relief suggests a strong therapeutic effect in the patient population studied.

The clinical evidence with HF10 SCS thus far suggests favorable outcomes relative to the published randomized clinical studies for back pain. Furthermore, leg pain relief seems to be comparable or perhaps better than traditional SCS when examining the magnitude of pain relief. Coupled with the lack of paresthesia, these results indicate that HF10 SCS provides significant, sustained pain relief without the consequences and clinical burden of managing paresthesia. We also note the remarkable improvement seen with patients who had previously failed SCS and/or PNFS. Previously, these patients were left with few options; such patients are commonly treated with opioids, which can lead to addiction, increase in pain syndromes, and a number of other issues [43].

With HF10 SCS therapy, we expect to see a number of changes in the typical management of the pain patient. Patients who had previously been considered as inappropriate candidates for traditional SCS, such as those with back pain or who had failed a SCS trial, may now go on to successful HF10 SCS therapy. Additionally, both the patient and physician benefit from the changes in the operating room. The HF10 SCS procedure is straightforward and more predictable in terms of time duration; leads can be implanted near the anatomical midline rather than the physiological midline, the patient can remain under sedation, and there is no need for extensive patient–physician feedback in the operating room.

Despite these results, opportunities to further optimize HF10 may exist. Such opportunities include duty cycling, which may further improve efficacy. Another opportunity may be to better understand patient selection through larger studies.

The main limitation of the discussed data is that they were from observational studies without a concurrent control group or from data review. However, the methodology and execution of the European study (large sample size, multicenter, long duration, and low dropout rate) reduce the risk of overestimating the magnitude of the treatment effect [28]. Despite the limitation, the data contribute significantly to our body of knowledge and expectations of clinical benefit.

It is worthwhile noting the results from a 33-patient, randomized, two-period, cross-over study on 5-kHz SCS [44]. The authors concluded that the 5-kHz SCS was equivalent to sham for the primary outcome (improvement of Patient Global Impression of Change). A notable limitation from the study is that enrolled patients were traditional SCS users. The authors wondered “whether patients naïve to stimulation would behave in a similar way.” The enrolled patients may have been conditioned to believe that paresthesia is required for pain relief. Also, programming in the 5-kHz study was based on paresthesia mapping, while HF10 programming is based on patient's response to pain relief. These limitations, as well as the difference in frequencies, may account for the different results.

Overall, we believe that HF10 SCS may have a significant impact on a physician's ability to treat selected patients in pain and may offer an improved patient experience with the HF10 SCS therapy.

## Conclusions

We have described an innovative approach to SCS, HF10 SCS, which uses 10-kHz high-frequency stimulation to provide pain relief without paresthesia and with more predictable procedure times. Data from multiple sources, including a multicenter, prospective, clinical trial, show that the therapy appears to provide substantial back pain relief and may provide improved pain relief in other body areas and for complex pain, even for patients who have previously failed SCS and/or PNFS. This therapy may change the way pain patients are treated with neuromodulation, potentially moving SCS to a place earlier in the treatment continuum.
